# Salesperson Self-Regulated Learning and Online Customers’ Patronage: An Ambidexterity Perspective

**DOI:** 10.3389/fpsyg.2021.795899

**Published:** 2021-12-01

**Authors:** Bing Han, Hua Fan

**Affiliations:** ^1^Shanghai University of International Business and Economics, Shanghai, China; ^2^School of Business and Management, Shanghai International Studies University, Shanghai, China

**Keywords:** exploratory learning, e-loyalty, patronage intention, patronage behavior, polynomial regression

## Abstract

Although the roles of exploratory and exploitative learning as alternative sales skills have been documented, there is not yet a clear consensus, and empirical evidence in the online sales context is lacking. In addition, existing studies have tended to examine the two activities in parallel, without looking into the dyadic situation of balanced or imbalanced exploratory-exploitative learning. Grounded in the WeChat business context, this study explores how online sales agents’ balanced and imbalanced ambidextrous learning influence customers’ e-loyalty and, in turn, their patronage intention and behavior. Polynomial regression and response surface analysis are performed on 226 dyads, and the results support the hypothesized balance effect. Further, asymmetrical imbalance effects are identified, with customers exhibiting higher e-loyalty and better patronage outcomes when online sales agents adopt more exploitative learning than exploratory learning. This study helps improve understanding of the efficiency of personal selling in a virtual context.

## Introduction

Galvanized by the thriving popularity of social networking, companies are increasingly relying on social media tools to sell products and services (Yang et al., 2016). In China, marketing on WeChat is an emerging way of promoting products and services. Over 10 million companies are deploying WeChat salespersons, and WeChat business accounts have reached total sales of over 150 billion RMB (Yang et al., 2016; Lien et al., 2017). Although the roles of salesperson exploitative and exploratory learning in triggering sales performance have been documented (e.g., Katsikeas et al., 2018), a still-unanswered question that has great relevance to WeChat business providers is how the influence of salesperson self-regulation on customers’ patronage differs when the salesperson is online vs. offline.

Salesperson exploratory learning involves “searching for, experimenting with, and discovering new selling techniques and skill sets that help improve sales performance” (Katsikeas et al., 2018, p. 50). In contrast, a salesperson engaging in exploitative learning “adheres to proven existing selling techniques and skill sets that leverage known knowledge and capabilities to enhance performance” (Katsikeas et al., 2018, p. 50). The exploratory style adds variety to the customer experience and plants new knowledge within customers’ memories (Seo et al., 2015). However, exploitative learning can help create a sense of reliability in the customer experience (Kane and Alavi, 2007). Therefore, salesperson exploratory and exploitative learning both play a pivotal role in shaping customers’ judgments and influencing sales performance (Katsikeas et al., 2018).

Although there is a large body of literature on the effects of exploitative and exploratory learning, a close review of this research in the marketing realm reveals three deficiencies (see Table 1). First, exploitative and exploratory learning have been examined in both online and offline contexts (i.e., at the inter-firm and interpersonal levels, respectively), but no study has integrated individual exploitative-exploratory learning within the online sales context. Online selling is a new e-business model, on which the development of customer relationships is distinguished from that in offline business, and even from traditional e-business (Yang et al., 2016). Although online selling provides users convenience in real-time communication, some issues such as decreasing face-to-face communications, addiction on the instant messaging service, and the stress of 24 h stand-by will negatively impact interpersonal interactions and social development (Lien et al., 2017). Therefore, it is necessary to know how individual learning works on the new online business platform. Second, although many studies have examined the outcomes of individual exploratory or exploitative learning, such as salesperson performance (Katsikeas et al., 2018), existing and new product sales (Van der Borgh and Schepers, 2018), service-sales ambidexterity (Yu et al., 2015), and firms’ profit margins (DeCarlo and Lam, 2016), there is still a dearth of research on the synergy effects between exploratory and exploitative learning. Third, the roles of exploration and exploitation—especially balanced exploratory-exploitative learning—in triggering performance outcomes are not clearly conclusive.

**TABLE 1:** Selected studies on exploratory and exploitative learning.

			**Type of learning and its link to performance outcomes**		
**Unit of analysis**	**Research context**	**Study**	**Exploratory learning**	**Exploitative learning**	**Balanced exploratory- exploitative learning**
• Firm level	• Offline context • Marketing strategy	Kyriakopoulos and Moorman, 2004	• New product financial performance (−)	• New product financial performance (+)	
		Auh and Menguc, 2005	• Effectiveness and efficiency firm performance (+)	• Effectiveness and efficiency firm performance (+)	
		Vorhies et al., 2011	• Customer-focused marketing capabilities (+)	• Customer-focused marketing capabilities (+)	• Customer-focused marketing capabilities (−)
		Sok and O’Cass, 2015			• Customer perceived service quality (0)
		Gualandris et al., 2018			• Suppliers’ efficiency (+) • Suppliers’ product innovation (+) • Buyer financial performance (+)
	• Offline context • New product development	Atuahene-Gima, 2005	• Radical innovation (+) • Incremental innovation (−)	• Radical innovation (−) • Incremental innovation (+)	
		Atuahene-Gima and Murray, 2007	• New product performance (+)	• New product performance (−)	• New product performance (−)
		Li et al., 2010	• New product development performance (∩)	• New product development performance (∩)	• New product development performance (−)
		Yannopoulos et al., 2012	• New product performance (+)	• New product performance (0)	
		Mu, 2015	• New product development performance (+)	• New product development performance (+)	
		Lee et al., 2017			• New product development performance (+)
	• Online context • Information technology	Lee et al., 2015			• Operational ambidexterity (+) • Organizational agility (+)
		Tai et al., 2019			• Operational support (+) • Strategic decision support (0)
		Benitez et al. (2018)	• Operational competence (+)	• Firm performance (+)	
• Individual level	• Offline context • Personal selling	Van der Borgh and Schepers, 2014	Task autonomy (−)	• Task autonomy (+)	Research Gap 2 Effects of individual level exploratory-exploitative learning balance and imbalance
		DeCarlo and Lam, 2016	• Hunting orientation (+) • Farming orientation (0)	• Hunting orientation (0) • Farming orientation (+)	
		Yu et al. (2015)	• Service-sales ambidexterity (0)	• Service-sales ambidexterity (−)	
		Van der Borgh et al., 2017	• Target obtainment with new products (+)	• Target obtainment with existing products (+)	
		Van der Borgh and Schepers, 2018	• Managerial overall performance evaluation (+) • New product selling performance (+)	• Effort to sell new products (−)	
		Katsikeas et al., 2018	• Salesperson performance (+)	• Salesperson performance (+)	
Research Gap 1 Individual level analysis of exploratory and exploitative learning under online sales context			Research Gap 3 Underlying mechanism for the conflicting influences of exploratory-exploitative behaviors		

*Studies on the organizational learning-performance relationship from the same data set are reported once. (+) denotes a positive relationship, (0) denote a non-significant relationship, (−) denotes a negative relationship, and (∩) denotes an inverted-U shaped relationship.*

To address these research deficiencies, this study has three aims. First, it focuses on personal selling behaviors in the context of WeChat business services, which seamlessly connect traditional e-commerce and social media communications and facilitate frequent and active real-time interpersonal interactions (Yang et al., 2016; Lien et al., 2017). Second, drawing on regulatory focus theory and ambidexterity theory, this study analyzes the effects of online salespersons’ balanced and imbalanced exploitative-exploratory learning on customers’ e-loyalty. Finally, this study identifies e-loyalty and patronage intention as key mediators that link (im)balanced exploratory-exploitative learning to customers’ ultimate patronage behavior. This study makes three contributions. First, it integrates the personal selling (i.e., online salesperson) and mobile marketing (i.e., WeChat business service) perspectives, thus helping to bridge organizational learning studies adopting different units of analysis (i.e., individual and online research contexts). Second, it contributes to the sales literature by differentiating the conditions of balanced and imbalanced exploratory-exploitative selling. Finally, it contributes to ambidexterity research by articulating the mechanisms through which personal selling ambidexterity influences performance; in so doing, it helps reconcile discordant findings on the link between salesperson ambidextrous selling activities and performance outcomes.

## Theoretical Background and Hypotheses

### Ambidexterity Theory, Regulatory Focus Theory, and Self-Regulated Learning

Ambidexterity theory argues that exploration and exploitation, when balanced or combined, form a unique advantage that can generate sustained financial success (e.g., March, 1991; Gibson and Birkinshaw, 2004; Choi and Lee, 2015). The concepts of exploration and exploitation are originally embedded in organizational behavior research; the former emphasizes flexibility and variability and refers to activities such as experimentation, searching, and risk taking, while the latter centers on efficiency and reliability and involves activities such as implementation, refinement, and execution (Levinthal and March, 1993). Although exploitation and exploration have different goals and require different competencies (Levinthal and March, 1993), they may still be accomplished together to obtain certain outcomes (Gibson and Birkinshaw, 2004). In particular, an organization or individual entity can be regarded as ambidextrous when it balances or combines exploitation and exploration; balancing the two allows it to achieve and maintain an equivalent focus on the two activities, while combining them involves achieving and maintaining a high pursuit of both (Gualandris et al., 2018).

Noting the scarcity of research on personal exploration and exploitation, Katsikeas et al. (2018), drawing on regulatory focus theory, conducted a pioneering study on salesperson exploitative and exploratory learning. The main proposition of regulatory focus theory is that people use two self-regulatory behaviors to achieve goals, namely, promotion-focused and prevention-focused behaviors (Higgins, 2002; Katsikeas et al., 2018). Following their definition, this study defines *salesperson exploratory learning* as an online salesperson’s self-regulated promotion-focused behavior that focuses on “experimenting with, searching for, and discovering novel, creative, and innovative selling techniques” (Katsikeas et al., 2018, p. 49). Exploratory learning is associated with long-term payoffs from selling, the exploration of new activities, the acceptance of uncertainty, and a higher willingness to take risk (Van der Borgh and Schepers, 2014). Thus, exploratory learning concentrates on avoiding faults of omission (i.e., missing a potential sales opportunity) and trying new sales skills (DeCarlo and Lam, 2016).

In contrast, *salesperson exploitative learning* refers to an online salesperson’s self-regulated prevention-focused behavior that “enhances productivity and efficiency by adhering to proven methods of selling and leveraging existing knowledge and experience, resulting in minimal deviation from routine selling” (Katsikeas et al., 2018, p. 49). Whereas exploration adds variety to experience, exploitation creates reliability in experience and concentrates on the implementation, diffusion, improvement, and reuse of current knowledge (Kane and Alavi, 2007; Seo et al., 2015). Therefore, exploitative learning focuses on exploiting existing activities for the accomplishment of short-term goals and the maintenance of the *status quo* (Van der Borgh and Schepers, 2014), with an emphasis on avoiding faults of commission (i.e., making mistakes), sticking to proven selling tactics, and enhancing protection (Katsikeas et al., 2018).

### (Im)balanced Exploratory and Exploitative Learning and Customers’ E-Loyalty

We apply the tenets of ambidexterity theory to differentiate learning balance from learning imbalance. Specifically, online salespersons can achieve the *balanced* version of ambidexterity by putting an equivalent emphasis on and adopting comparable levels of exploratory and exploitative selling skills. They can experience a sense of balance by adopting similar (either high or low) levels of new and existing selling skills. Conversely, they experience imbalance when one type of selling skill starts to outweigh the other.

The balance or imbalance of online salespersons’ self-regulated learning has important consequences for customers’ e-loyalty. In the online business context, salespersons must shift from traditional and purely commercial selling approaches to a combination of both conservative and innovative selling (Yang et al., 2016). On the one hand, by emphasizing the importance of using both routine and novel selling skills, it is possible to raise online salespersons’ awareness of the drawbacks of relying on a single, monotonous selling method (Van der Borgh and Schepers, 2014). Increasing their awareness of both selling tactics will guide them to better allocate their resources and enhance their outcomes (Gibson and Birkinshaw, 2004). On the other hand, online salespersons’ prior success in using routine selling approaches can help reveal customers’ needs and potential purchase opportunities, such that customers are likely to respond favorably to the combination of routine and novel approaches (Yu et al., 2015). Therefore, balanced ambidexterity can increase performance (Van der Borgh et al., 2017), with customers not only mentally adhering to the online business relationship but also repeatedly visiting and purchasing from the online salespersons.

However, ambidexterity theory also suggests that an imbalance in online salespersons’ self-regulated learning will dampen customers’ e-loyalty. Divergent interpretations of self-regulated learning can blur expectations and impede the allocation of cognitive efforts to each learning style (Hobfoll, 2002). When confronted with mixed demands as to the use of exploratory vs. exploitative selling skills, online salespersons will suffer from role ambiguity and role conflict (Van der Borgh and Schepers, 2014; Yu et al., 2015). Unclear goal focus and conflicting demands may hinder online salespersons’ task outcomes (Locke and Latham, 2002), resulting in adverse effects for customers, such as strained business relationships and customers’ estrangement (Aksin et al., 2007). Thus, we expect:

*H1: The greater the balance between an online salesperson’s exploratory and exploitative learning, the higher customers’ e-loyalty.*

### Differentiating the Two Scenarios of Exploratory-Exploitative Learning Balance

Ambidexterity theory suggests that the *combined* version of ambidexterity can be achieved by frequently and simultaneously implementing both exploration and exploitation, with the interaction of these activities resulting in superior sales performance (Van der Borgh et al., 2017). In anonymous online business transactions, online salespersons’ exploitative learning is essential for meeting diverse customer needs in a standardized and safe manner (Van der Borgh et al., 2017). To increase sales effectiveness, the incorporation of creative and novel selling skills into such transactions should be done carefully, not in a way that confronts customers with a completely new experience (Van der Borgh and Schepers, 2018).

Explorative learning, i.e., the taking of initiative in selling products and services, is also critical in online business transactions (Belschak et al., 2010). Presenting customers with new selling approaches along with proven selling skills may help online salespersons accentuate the benefits of their services and products (Van der Borgh and Schepers, 2018). Therefore, compared with equally low levels of exploratory and exploitative learning, customers are more inclined to accept and commit to a sales approach in which online salespersons’ exploitative and exploratory learning are frequently performed together—i.e., combined ambidextrous learning. Thus, we hypothesize:

*H2: Customers exhibit greater e-loyalty when an online salesperson balances exploitative and exploratory learning at higher levels than when the salesperson balances exploitative and exploratory learning at lower levels.*

### Differentiating the Two Scenarios of Exploratory-Exploitative Learning Imbalance

When online salespersons can achieve neither balanced ambidextrous learning nor combined ambidextrous learning, two situations are plausible. In the first situation, they rely more on routine selling skills than on novel techniques. Exploitative learning focuses on sticking to existing solutions, making incremental upgrades/modifications, and forming ideas within a conventional framework (Seo et al., 2015). As a risk prevention-focused behavior, exploitative learning enhances online salespersons’ performance through adherence to proven skill sets and selling techniques (Katsikeas et al., 2018), such as standardized and defined service procedures (Yu et al., 2015). Therefore, online salespersons are likely to deploy less exploratory and more exploitative learning because it is the safer type of selling behavior (Higgins, 2002; Avnet and Higgins, 2006). Online customers, in turn, are likely to favor such proven selling approaches, because customers are often reluctant to accept new approaches and tend to exhibit passive and reactive behavior (Van der Borgh et al., 2017).

In the second situation, online salespersons rely more on creative and innovative selling skills than on proven techniques. In this case, they are required to gain greater knowledge and actively participate in non-routine processes to identify sales opportunities (Yu et al., 2015). On the one hand, the time and effort they devote to learning, testing, and discovering innovative and creative selling techniques engender risk and ambiguity (Katsikeas et al., 2018), with their efforts having uncertain returns in terms of sales outcomes (Yu et al., 2015). On the other hand, an overemphasis on exploratory learning might push online salespersons into radical knowledge-searching behaviors that depart from established directions (Seo et al., 2015), such as digging into customers’ purchase history and preferences and leaking their personal information. As radical selling increases, online customers are likely to undertake countermeasures to protect their privacy (Yao and Cao, 2017). Therefore, when the implementation of exploratory selling exceeds that of exploitative selling, both online salespersons and customers might be reluctant to dedicate themselves to the dyadic relationship. Thus, we hypothesize:

*H3: Customers exhibit less e-loyalty when an online salesperson implements more exploratory learning than exploitative learning than when the salesperson implements more exploitative learning than exploratory learning.*

### E-Loyalty and Patronage Intention as Chain-Mediators of the (Im)balance Effect on Patronage Behavior

Customers’ e-loyalty is a combination of their attitudinal and behavioral propensity (Kim et al., 2018; Kingshott et al., 2018), and customers’ patronage similarly represents their positive attitudes and behaviors toward the salesperson (Blut et al., 2018). Research on loyalty and its outcomes has illuminated various positive consequences of loyalty to salespeople, such as the extension of loyalty to the selling firm, customer willingness to pay a price premium, higher selling effectiveness, and sales growth (e.g., Reynolds and Beatty, 1999; Palmatier et al., 2007). Therefore, when online salespersons frequently and simultaneously implement both exploration and exploitation, balanced and combined ambidextrous learning can trigger high levels of customer loyalty, which, in turn, results in better customer attitudes toward the salespersons and a higher purchase volume.

Given that we have hypothesized the effects of balanced and imbalanced exploratory-exploitative learning on customers’ e-loyalty and the aforementioned relationship between e-loyalty and customers’ patronage, we expect e-loyalty and patronage intention to play a chain-mediating role in the (im)balance effects and customers’ patronage behavior. This conduit highlights that learning ambidexterity, both balanced and combined, is important to online business providers because it can influence customer attitudes and purchase decisions through customers’ improved relationships with their salespersons. Thus, we hypothesize:

*H4: Customers’ e-loyalty and patronage intention play a chain-mediating role in the relationship between online salespersons’ (im)balanced exploratory-exploitative learning and customers’ patronage behavior.*

## Materials and Methods

### Research Design

WeChat is the dominant instant messaging communication platform in China (Lien and Cao, 2014), and the WeChat business model represents an ideal online marketing research context because it integrates traditional e-commerce activities and social media communications (Yang et al., 2016). We collected data from the staff and customers of a WeChat business service provider that focuses on selling cosmeceuticals. Separate questionnaires were designed for online salespersons and their customers independently to minimize common method bias.

We collected a sample of 300 online salespersons from the WeChat business provider. The online salespersons were first asked via phone whether they wanted to participate in the survey. All of them agreed, and we obtained their names and e-mail addresses. We then e-mailed the survey questionnaires to them, asking them to identify a customer they had dealt with and provide his/her contact information. Three months later, the matched questionnaires were sent to the named customers. The qualifying customer respondents were carefully chosen according to three criteria recommended by Lien et al. (2017). First, only those who had made at least one purchase decision in the previous month were qualified to take part in the survey. Second, only residents of Beijing, Shanghai, Guangzhou, and Shenzhen were selected, as 93% of people living in these cities are registered WeChat users (Lien et al., 2017). Third, this study identified younger generations, who are more familiar with cosmeceutical products, as the target group. The final sample consisted of 226 paired transaction relationships.

### Measurement and Validity

In response to the call of Katsikeas et al. (2018) for an objective performance measure of salesperson learning outcomes, we used the objective purchase amount to measure the *patronage behavior* of a given customer. For other variables, all of our measurements were adapted from previous research. Table 2 reports the sources of the measurement items. The following control variables were included: (1) business providers’ e-service quality, (2) alternative providers’ attractiveness, (3) customers’ trust perception, and (4) real-time interactivity experience. Of the above variables, studies have shown that the first two influence customers’ e-loyalty (Kim et al., 2018; Kingshott et al., 2018) and the latter two influence online customers’ patronage (Keeling et al., 2010; Etemad-Sajadi, 2016). Self-regulated learning information was collected from the WeChat business salespersons. Customers’ e-loyalty and patronage and the control variables were collected from the online customers.

**TABLE 2:** Construct reliability and validity.

**Patronage intention (Keeling et al., 2010; Etemad-Sajadi, 2016; Time 2; α = 0.894; CR = 0.896; AVE = 0.743)**	**Factor loadings**
(1) Visiting this online salesperson increases my desire to make business with the company.	0.854
(2) This online salesperson gives me the impression that making business with this company will be positive.	0.930
(3) It is likely for me to buy from, recommend, and revisit this online salesperson.	0.797
**E-loyalty (Kingshott et al., 2018; Time 2; α = 0.907; CR = 0.908; AVE = 0.766)**	
(1) We intend to continue using this online salesperson’s e-commerce services.	0.839
(2) We will continue to use this online salesperson’s e-commerce services for all future transactions.	0.870
(3) We will recommend this online salesperson’s e-commerce services to others.	0.915
**Exploratory learning (Katsikeas et al., 2018; Time 1; α = 0.893; CR = 0.894; AVE = 0.629)**	
(1) I search for novel information and ideas that enable me to learn new sales techniques.	0.748
(2) I discover new selling techniques that take me beyond my current knowledge, skills, and abilities in improving my performance.	0.723
(3) I engage in learning new selling skills and knowledge that help me look at customers’ problems in a different light.	0.823
(4) I explore novel and useful approaches that I can use to respond to customers’ needs and wants in the future.	0.852
(5) I focus on learning new knowledge of selling techniques that involve experimentation and potential risk of failure.	0.813
**Exploitative learning (Katsikeas et al., 2018; Time 1; α = 0.713; CR = 0.862; AVE = 0.555)**	
(1) I adhere to sales techniques that I can implement well to ensure productivity rather than those that could lead me to implementation mistakes.	0.740
(2) I implement my proven approaches to leverage my existing knowledge and experience in selling to customers.	0.748
(3) I adopt sales techniques that suit well to my current knowledge and experience.	0.775
(4) I execute those sales techniques that are aligned well with my selling routines.	0.693
(5) I prefer undertaking sales tasks with little variation in my performance compared to sales tasks with handsome rewards but with risks involved.	0.767
**E-service quality (Kingshott et al., 2018; Time 2; α = 0.917; CR = 0.920; AVE = 0.700)**	
(1) This company provides a high level of e-commerce service quality.	0.769
(2) This company provides user-friendly e-commerce facilities.	0.901
(3) This company’s e-commerce facilities are reliable.	0.915
(4) This company’s e-commerce facilities enable quick information.	0.870
(5) This company’s e-commerce has flexibility to fulfill our specific needs.	0.708
**Alternative attractiveness (Kim et al., 2018; Time 2; α = 0.955; CR = 0.955; AVE = 0.877)**	
(1) If I need to change the current provider, there are other good providers to choose from.	0.928
(2) I would feel more satisfied with the services of another provider as compared to the current provider.	0.948
(3) I would be more satisfied with price plans of another provider as compared to the current provider.	0.933
**Online real-time interactivity (Etemad-Sajadi, 2016; Time 2; α = 0.877; CR = 0.916; AVE = 0.687)**	
(1) This company allows me to interact with it in order to receive information.	0.818
(2) This company has interactive features to meet my needs.	0.873
(3) This company allows to easily find the desired information without having to call the company.	0.905
(4) This company allows to easily find the desired information without having to write an email to the company.	0.795
(5) The interaction with this company is efficient.	0.742
**Trust perception (Keeling et al., 2010; Time 2; α = 0.838; CR = 0.839; AVE = 0.567)**	
(1) I would believe the information given from this company.	0.761
(2) I would trust the payment process of this company.	0.815
(3) I would be confident that my order was correct.	0.712
(4) I would use the recommendations from this company.	0.720
*Notes:*	

*α, Cronbach’s alpha; CR, composite reliability. AVE, average variance extracted.*

We performed a confirmatory factor analysis, and the results showed that our data had an adequate fit to the measurement model (χ^2^ = 626.932, *df* = 467; RMSEA = 0.039; CFI = 0.964; TLI = 0.959). As Table 2 shows, the estimates of Cronbach’s alpha and composite reliability (CR) were higher than 0.7, indicating good reliability (Fornell and Larcker, 1981). The values of the average variance extracted (AVE) were above 0.5, exhibiting good convergent validity (Fornell and Larcker, 1981). The AVE value of each variable was larger than all of the correlations among constructs, indicating adequate discriminant validity. In addition, we followed Lindell and Whitney’s (2001) procedure to examine common method variance (CMV). As seen in Table 3, the lowest positive pairwise correlation was 0.005. We adjusted the correlations based on the lowest positive pairwise correlation, which can be regarded as a valid indicator of CMV (Lindell and Whitney, 2001). The results showed that there was no significant correlation lost, which suggests that CMV is not likely to be a serious issue.

**TABLE 3:** Descriptive statistics and Pearson’s Correlation Matrix (*N* = 226).

**Variables**	**1**	**2**	**3**	**4**	**5**	**6**	**7**	**8**	**9**
(1) Patronage intention									
(2) Patronage behavior	0.469**								
(3) Exploitative learning	0.011	0.160*							
(4) Exploratory learning	−0.108	0.141*	0.612**						
(5) Customers’ e-loyalty	0.330**	0.169*	0.630**	0.176**					
(6) Trust perception	0.220**	0.168*	0.065	0.149*	0.053				
(7) Online real-time interactivity	0.109	0.179**	0.005	0.141*	−0.074	0.193**			
(8) Alternative attractiveness	0.036	0.109	−0.011	0.015	0.037	−0.069	0.084		
(9) E-service quality	−0.007	0.022	−0.023	−0.017	0.060	0.015	−0.116	0.130	
Mean	4.527	4.627	4.902	4.632	4.566	3.724	5.251	4.378	4.011
*S.D.*	0.748	0.961	1.509	1.349	0.682	0.817	1.158	1.515	1.487

*Correlation is significant at the 0.05 level (two-tailed). **Correlation is significant at the 0.01 level (two-tailed).

### Analytical Approach

Multivariate regression analysis has limitations in accurately detecting the combined and balanced effects of exploration and exploitation (Lee et al., 2017), and there are growing appeals to avoid the methodological problems created by difference scores (Gao and Fan, 2021). Against this backdrop, the polynomial regression analysis introduced by Edwards and Parry (1993) represents the latest in a line of research methods designed to calculate and assess the effect of (im)balance. In our polynomial modeling, the mediator variable (e.g., customers’ e-loyalty) was regressed on the control variables, online salespersons’ exploratory learning (EPR) and exploitative learning (EPT), and three higher-order effects (i.e., EPR^2^, EPT^2^, and EPR × EPT) after scale-centering both EPR and EPT (see Table 4). Following the steps of response surface analysis (Edwards and Parry, 1993), the estimated coefficients were used to calculate the slopes and curvatures. We calculated the parameters along the balance (EPR = EPT) and imbalance (EPR = −EPT) lines as the balance slope (EPR + EPT), the balance curvature (EPR^2^ + EPR × EPT + EPT^2^), the imbalance slope (EPR − EPT), and the imbalance curvature (EPR^2^ − EPR × EPT + EPT^2^). To test the direct effects of (im)balanced self-regulated learning on customers’ e-loyalty (H1-3), we used the coefficients’ significance of the slopes and curvatures.

**TABLE 4:** Polynomial regression results.

	**E-loyalty**		**Patronage intention**			**Patronage behavior**		
**Variables**	**Model 1**	**Model 2**	**Model 3**	**Model 4**	**Model 5**	**Model 6**	**Model 7**	**Model 8**
Constant	4.394**	4.225**	3.344**	3.802**	1.899**	2.864**	3.041**	1.157^†^
**Control variables**								
Trust perception	0.060	0.036	0.193**	0.152**	0.136**	0.172*	0.126^†^	0.032
Online real-time interactivity	−0.051	−0.022	0.041	0.042	0.052	0.120*	0.085^†^	0.055
Alternative attractiveness	0.020	0.023	0.023	0.021	0.010	0.066	0.063	0.052
E-service quality	0.020	0.023	−0.005	−0.018	−0.028	0.015	0.019	0.034
**Polynomial terms**								
Exploitative learning (EPT)	0.366**		0.126*	−0.039		0.010	−0.021	
Exploratory learning (EPR)	−0.138**		−0.167**	−0.105*		0.040	0.129^†^	
EPT^2^	−0.041*		−0.157**	−0.138**		−0.004	0.093**	
EPT × EPR	0.102**		0.379**	0.333**		0.209**	−0.024	
EPR^2^	−0.090**		−0.190**	−0.150**		−0.086*	0.025	
**Mediators**								
E-loyalty			0.451**			0.141		
Patronage intention					0.652**			
*R* ^2^	0.014	0.508	0.055	0.373	0.456	0.062	0.176	0.322
Δ*R*^2^	0.494**		0.318**	0.083**		0.114**	0.146**	
**Balance line (EPR = EPT)**								
Slope	0.228**		−0.041	−0.143**		0.050	0.108*	
Curvature	−0.029		0.031	0.044*		0.119**	0.094**	
**Imbalance line (EPR = −EPT)**								
Slope	0.504**		0.293**	0.066		−0.030	−0.150	
Curvature	−0.233**		−0.726**	−0.621**		−0.299**	−0.142	

Unstandardized regression coefficients are reported. ^†^*p* < 0.10, *p < 0.05, **p < 0.01. Two-tailed tests.

Following the block variable approach (Gao and Fan, 2021), we tested the indirect effects of (im)balanced self-regulated learning on customers’ patronage (H4). A block variable was computed as a weighted composite score by multiplying the raw data by the polynomial coefficients. Then, both the mediation variables (i.e., customers’ e-loyalty and patronage intention) and the outcome variables (i.e., patronage behavior) were regressed on the block variable to obtain the path coefficients. By multiplying the path from the block variable to customers’ e-loyalty (“α” path) by the path from e-loyalty to patronage intention (“β” path) and by the path from patronage intention to patronage behavior (“γ” path) (see Table 5), we computed the indirect effects. A bootstrapping technique (10,000 samples) was applied to compute the bias-corrected confidence intervals (e.g., Gao and Fan, 2021).

**TABLE 5:** Indirect effect of exploratory-exploitative learning balance (imbalance) on patronage behavior.

	**Block variable to e-loyalty**	**E-loyalty to patronage intention**	**Patronage intention to patronage behavior**	**Indirect effect of learning (im)balance**
**Variables**	**“α” path**	**“β” path**	**“γ” path**	**“αβγ”**
Unstandardized results	1.002**	0.450**	0.653**	0.295**
90% bias-corrected bootstrapped confidence intervals for the indirect effect				[0.218, 0.384]
95% bias-corrected bootstrapped confidence intervals for the indirect effect				[0.205, 0.402]
99% bias-corrected bootstrapped confidence intervals for the indirect effect				[0.181, 0.440]
Standardized results	0.705**	0.411**	0.508**	0.147**
90% bias-corrected bootstrapped confidence intervals for the indirect effect				[0.110, 0.184]
95% bias-corrected bootstrapped confidence intervals for the indirect effect				[0.103, 0.192]
99% bias-corrected bootstrapped confidence intervals for the indirect effect				[0.089, 0.206]

**p < 0.01.

## Results

**FIGURE 1:**
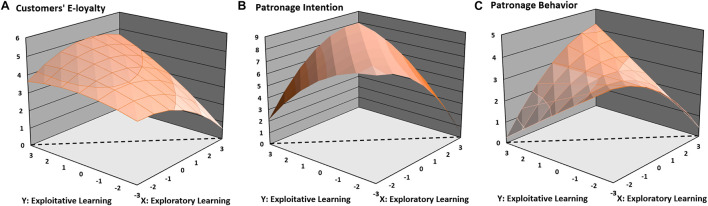
Hypothesized response surface graphs. The line of imbalance is depicted with the dotted line along the floor of the graph.

H1 predicted a balance effect, such that the greater the balance between an online salesperson’s exploratory and exploitative learning, the higher a customer’s e-loyalty. This reflects a significant negative curvature for the imbalance line (EPR = −EPT). As illustrated in Figure 1A, the surface along the imbalance line curved downward (curvature [EPR^2^ − EPR × EPT + EPT^2^] = −0.233, *p* < 0.01), demonstrating that customers’ e-loyalty was higher when exploratory and exploitative learning were equivalent, and any deviations from the balance line (EPR = EPT) were associated with less e-loyalty. In sum, H1 was supported.

H2 predicted that customers’ e-loyalty is greater when exploratory and exploitative learning are balanced at a high level than when they are balanced at a low level. This reflects a significant positive slope for the balance line (EPR = EPT). As illustrated in Figure 1A, the slope of the balance line (EPR = EPT) was significant and positive (slope [EPR + EPT] = 0.228, *p* < 0.01), suggesting that the high-high balance condition was associated with higher e-loyalty than the low-low balance condition. These results suggest support for H2.

H3 predicted an asymmetrical imbalance effect such that customers’ e-loyalty is lower when an online salesperson implements more exploratory than exploitative learning. This reflects the significant negative slope of the imbalance line (EPR = −EPT). As illustrated in Figure 1A, the slope along the imbalance line (EPR = −EPT) was significant and positive (slope [EPR − EPT] = 0.504, *p* < 0.01), thus supporting H3.

**FIGURE 2:**
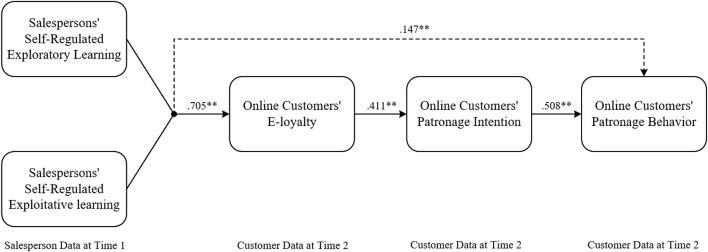
Hypothesized model for the current study and estimated standardized coefficients. → represents indirect paths via e-loyalty and patronage intention. → represents direct paths. ^∗^*p* < 0.05, ^∗∗^*p* < 0.01.

H4 predicted that the relationships between (im)balanced exploratory-exploitative learning and customers’ patronage behavior are mediated by customers’ e-loyalty and patronage intention. First, we computed three block variables with the estimated unstandardized coefficients of the five polynomial terms (EPR, EPT, EPR^2^, EPR × EPT, and EPT^2^) using e-loyalty (mediator), patronage intention (mediator), and patronage behavior (outcome variable) as dependent variables. The effect of (im)balanced exploratory-exploitative learning on customers’ e-loyalty (α = 1.002, *p* < 0.01) was significant (see Table 5). The paths between customers’ e-loyalty and patronage intention (β = 0.450, *p* < 0.01) and between customers’ patronage intention and patronage behavior (γ = 0.653, *p* < 0.01) were also significant and positive, as predicted. The indirect effect (i.e., the product of α, β, and γ) between (im)balanced exploratory-exploitative learning and customers’ patronage behavior that was carried through customers’ e-loyalty and patronage intention was 0.295. The 95% confidence intervals of the examined indirect path did not include 0 (lower bound = 0.205, upper bound = 0.402), supporting H4. As a supplemental analysis, we also calculated the indirect path via standardized regression coefficients and examined 90% as well as 99% confidence intervals for both the unstandardized and standardized indirect paths (see Table 5). For the two patronage variables, we also conducted *post hoc* analyses and created response surface graphs (see Figures 1B,C), supporting the balance/imbalance effects of exploratory-exploitative learning. We propose our conceptual framework and estimated the standardized coefficients in Figure 2.

## Discussion

Most ambidexterity studies are confined to the organizational research context. The importance of exploration-exploitation ambidexterity has been underemphasized in the personal selling literature (Katsikeas et al., 2018), especially in terms of the influence of salespersons’ (im)balanced exploitative and exploratory learning on the success of online interactions. Grounded in the WeChat business context, this study examines how online salespersons’ personal balance of exploratory and exploitative learning contributes to customers’ e-loyalty and patronage. The findings not only contribute new insights to mobile marketing research but also provide empirical evidence to the adaptive selling and ambidexterity literatures. The findings also have practical implications, offering guidance to online salespersons and managers at companies involved in online marketing and concerned with customer relationship management.

### Theoretical and Managerial Implications

By exploring salespersons’ personal balance of exploratory and exploitative learning and how these balance effects operate in the online sales context, this study connects two research domains: mobile marketing and personal selling. Previous studies have highlighted the roles of ambidexterity in organizations’ marketing strategy implementation, new product development, and information technology application (e.g., Mu, 2015; Sok and O’Cass, 2015; Benitez et al., 2018). However, scarce attention has been paid to individual ambidextrous learning (Katsikeas et al., 2018), especially online salespersons’ exploratory and exploitative learning. Our focus on the WeChat business context answers the call of Katsikeas et al. (2018) for generalizable assessments of salesperson exploratory and exploitative learning under different research contexts. The results of this study help paint a vibrant picture of personal selling in mobile marketing settings.

This study also extends the adaptive selling literature by proposing possible synergy effects of individual selling skills and offering new evidence on the effects of balanced and combined exploratory-exploitative learning. Exploitation and exploration are traditionally conceived as isolated actions (March, 1991), and ambidexterity studies at the individual level of analysis have only examined exploration and exploitation effects separately (e.g., Yu et al., 2015; DeCarlo and Lam, 2016; Katsikeas et al., 2018). The findings of this study indicate that exploratory and exploitative learning are not independent of one another, and online salespersons can effectively stir positive attitudes in customers if only they can balance the two approaches. In this respect, our study agrees with the notion of Van der Borgh et al. (2017) that “exploration and exploitation balance can be achieved and, over time, increase performance for both goals” (p. 333). Thus, our study provides new insights regarding the adaptive use of individual selling skills.

Further, our study contributes to ambidexterity research by revealing the underlying mechanisms through which individual ambidextrous learning influences customers’ patronage. Studies have uncovered significant performance outcomes related to exploration and exploitation within both organizational and individual research contexts, such as new product financial performance, task autonomy, and sales-service ambidexterity (e.g., Kyriakopoulos and Moorman, 2004; Van der Borgh and Schepers, 2014; Yu et al., 2015). However, the empirical results of these studies reflect a lack of consensus on the effectiveness of exploration and exploitation in predicting performance outcomes. By introducing online customers’ e-loyalty and patronage intention as critical chain-mediators, this study sheds light on the paths from individual ambidextrous learning to its performance outcomes and reconciles the conflicting findings regarding exploratory-exploitative behaviors. By introducing appropriate mediators that are more proximal to sales outcomes, this study also answers the call of Katsikeas et al. (2018) for “a more robust and rigorous test […] to include cognition-, attitude-, and behavior-related mediators” (p. 67).

The findings of this study also provide valuable insights to practitioners in online marketing sectors, especially mobile marketing providers. First, the findings highlight that balanced exploratory-exploitative learning is consistently superior to imbalanced learning in online transaction interactions. Therefore, online salespersons should try to establish an ambidextrous selling orientation, rather than trading off between exploratory and exploitative selling skills. Exploitation and exploration, although distinct, are interdependent (Levinthal and March, 1993), and it is therefore essential for companies and salespersons to find ways to perform both and generate synergies. Second, the results suggest that when balanced ambidextrous selling is hard to achieve, online customers prefer exploitative over exploratory selling. Radically transforming traditional selling methods in the name of creativity can have unexpected negative consequences, including deterioration in sales and service levels and salesperson dissatisfaction (Aksin and Harker, 1999). Therefore, online salespersons should exercise caution in using innovative selling skills and would be well-advised to rely more on routine and proven techniques if their ambidextrous selling ability is limited. Third, this study emphasizes the sustainable bonds between customers’ attitudinal and behavioral outcomes. Given the chain-mediating route from online salespersons’ ambidextrous selling to customers’ ultimate purchase behavior, it is important to identify a customer’s emotional perceptions toward his/her corresponding salesperson. As such, online salespersons should pay attention to customers’ attitudes and feelings ahead of their purchase decisions, and firms’ training systems should concentrate on teaching sales personnel how to harvest customers’ e-loyalty.

### Limitations and Future Research

First, although this study adopted a time-lagged survey design by collecting questionnaires from customers 3 months after collecting questionnaires from online salespersons, the effect of ambidextrous learning across time is still unclear. It is possible that balancing exploration and exploitation activities over time is more effective than performing them simultaneously (Van der Borgh et al., 2017). Thus, future research may investigate the change in salesperson exploratory and exploitative learning over time (Katsikeas et al., 2018).

Second, our exclusive focus on successful business interactions may raise concerns about generalizability, as customers’ attitudinal and behavioral perceptions are relatively positive in such cases. In other words, online customers who did not identify with salespersons’ ambidextrous learning might have been excluded automatically from the sample collection. However, the non-significant relationships between online salespersons’ learning and customer patronage (see Model 8 in Table 4) suggest that customer behavior is not directly manipulated by ambidextrous learning, thus alleviating concerns over the uncollected sample. However, future studies could take other behavior-related variables as performance outcomes to capture customers’ negative feelings, emotions, and attitudes, such as customers’ migration behavior and firms’ multi-channel cannibalization.

Third, we collected questionnaires on the independent variables (i.e., exploratory and exploitative learning) and the dependent variables (i.e., customers’ e-loyalty and patronage) from online salespersons and their customers, respectively, helping to reduce CMV. However, salespersons’ self-reported selling skills might not exactly match customers’ perceptions, which could result in a response bias (Liu et al., 2018). Thus, future research should select online customers as the respondents for measures of self-regulated learning, because their perceptions of salespersons’ ambidextrous learning drive their loyalty and ultimate patronage. In addition, our sample consisted only of Chinese respondents. As research guided by Western thought might not be a perfect fit for the Chinese market, ambidextrous learning and online marketing practices may not be the same in China as they are in Western countries. Future studies should investigate the hypotheses of the present study in Western countries for comparison.

## Data Availability Statement

The raw data supporting the conclusions of this article will be made available by the authors, without undue reservation, to any qualified researcher.

## Author Contributions

BH and HF: conceptualization, methodology, validation, resources, writing—review and editing, and funding acquisition. BH: software, formal analysis, investigation, data curation, supervision, and project administration. HF: writing—original draft preparation and visualization. Both authors have read and agreed to the published version of the manuscript.

## Conflict of Interest

The authors declare that the research was conducted in the absence of any commercial or financial relationships that could be construed as a potential conflict of interest. The handling editor declared a shared affiliation with one of the authors BH at the time of the review.

## Publisher’s Note

All claims expressed in this article are solely those of the authors and do not necessarily represent those of their affiliated organizations, or those of the publisher, the editors and the reviewers. Any product that may be evaluated in this article, or claim that may be made by its manufacturer, is not guaranteed or endorsed by the publisher.
